# A review on the eco-epidemiology and clinical management of human granulocytic anaplasmosis and its agent in Europe

**DOI:** 10.1186/s13071-019-3852-6

**Published:** 2019-12-21

**Authors:** Ioana A. Matei, Agustín Estrada-Peña, Sally J. Cutler, Muriel Vayssier-Taussat, Lucía Varela-Castro, Aleksandar Potkonjak, Herve Zeller, Andrei D. Mihalca

**Affiliations:** 1 Department of Parasitology and Parasitic Diseases, Faculty of Veterinary Medicine, University of Agricultural Science and Veterinary Medicine Cluj-Napoca, Cluj-Napoca, Romania; 2 Department of Animal Health, Faculty of Veterinary Medicine, University of Zaragoza, Zaragoza, Spain; 3 School of Health, Sport & Bioscience, University of East London, London, UK; 4 Department of Animal Health, French National Institute for Agricultural Research, Maisons-Alfort, France; 5 Animal Health Department, NEIKER-Instituto Vasco de Investigación y Desarrollo Agrario, Bizkaia Science and Technology Park, Derio, Bizkaia Spain; 6 Department of Veterinary Medicine, Faculty of Agriculture, University of Novi Sad, Novi Sad, Serbia; 7 Emerging and Vector-borne Diseases Programme, European Centre for Disease Prevention and Control, Solna, Sweden

**Keywords:** HGA, *Anaplasma phagocytophilum*, Transmission, Prevalence, Clinical signs, Diagnosis

## Abstract

*Anaplasma phagocytophilum* is the agent of tick-borne fever, equine, canine and human granulocytic anaplasmosis. The common route of *A. phagocytophilum* transmission is through a tick bite, the main vector in Europe being *Ixodes ricinus*. Despite the apparently ubiquitous presence of the pathogen *A. phagocytophilum* in ticks and various wild and domestic animals from Europe, up to date published clinical cases of human granulocytic anaplasmosis (HGA) remain rare compared to the worldwide status. It is unclear if this reflects the epidemiological dynamics of the human infection in Europe or if the disease is underdiagnosed or underreported. Epidemiologic studies in Europe have suggested an increased occupational risk of infection for forestry workers, hunters, veterinarians, and farmers with a tick-bite history and living in endemic areas. Although the overall genetic diversity of *A. phagocytophilum* in Europe is higher than in the USA, the strains responsible for the human infections are related on both continents. However, the study of the genetic variability and assessment of the difference of pathogenicity and infectivity between strains to various hosts has been insufficiently explored to date. Most of the European HGA cases presented as a mild infection, common clinical signs being pyrexia, headache, myalgia and arthralgia. The diagnosis of HGA in the USA was recommended to be based on clinical signs and the patient’s history and later confirmed using specialized laboratory tests. However, in Europe since the majority of cases are presenting as mild infection, laboratory tests may be performed before the treatment in order to avoid antibiotic overuse. The drug of choice for HGA is doxycycline and because of potential for serious complication the treatment should be instituted on clinical suspicion alone.

## Background

Researchers interest on tick-borne pathogens (TBPs) has increased during the last decades with recognition of new agents, e.g. *Neoehrlichia mikurensis* and “*Candidatus* Anaplasma camelii” [1–3] and expansion of established tick-borne pathogens, driven by factors such as climatic changes and altered land use [4, 5]. TBPs dynamics, especially occurrence and abundance, are multifactorial, and strongly influenced by ecological interactions of tick species and their vertebrate hosts. The pivotal impact of climate change upon the geographical distribution of ticks, their abundance and host feeding patterns has become increasingly recognised [4–6] together with social changes, globalisation and intercontinental traveling of humans and animals influencing both the geographical distribution and abundance of ticks and pathogens [6].

Genus *Anaplasma* (Rickettsiales: *Anaplasmataceae*) is comprised of various species capable of causing disease among a variety of vertebrate hosts, including humans. The currently recognized species are *Anaplasma bovis*, *A. centrale*, *A. marginale*, *A. phagocytophilum*, *A. platys*, *A. ovis* and the more recently described *A. odocoilei* and *A. capra* [7–9]. These small pleomorphic Gram-negative bacteria (0.2–1.5 µm) are obligate intracellular microbes primarily transmitted by ticks [10]. *Anaplasma phagocytophilum*, the agent of granulocytic anaplasmosis, from a human perspective, is considered one of the most important species as a result of its zoonotic potential [11]. It is the etiological agent of tick-borne fever (TBF) in ruminants and of equine, canine and human granulocytic anaplasmosis (EGA, CGA and HGA, respectively) [7, 12]. Infections with *A. phagocytophilum* in animals are commonly reported in the northern hemisphere, being among the most widespread TBP in Europe [13]. Moreover, the geographical distribution of the pathogen and its main vector (*Ixodes ricinus*) are increasing in latitude and altitude [13] covering almost the entire territory of continental and Atlantic Europe.

Genetic diversity is being increasingly recognised amongst European strains of *A. phagocytophilum* demonstrated through phylogenetical analysis of genes such as *groEL* (chaperone protein encoding gene) [14–16], *ankA* (cytoplasmic protein antigen with ankyrin repeats encoding gene) [17–21] and *msp4* (major surface protein 4 encoding gene) [22]. *GroEL* gene is one of the two genes belonging to the heat shock operon *groESL* which encodes for the expression of highly conserved heat-shock proteins [23]. *GroEL* gene is considered a suitable marker to discriminate between *A. phagocytophilum* ecotypes distinguishing variants of different pathogenicity or geographical origin better than the *16S* RNA locus [16]. The *ankA* gene encodes a protein which has repeated ankyrin motifs. It might be a virulence factor and it has been hypothesized to be involved in host adaptation underlying diversifying selection [19, 21, 23]. Sequencing *ankA* distinguishes variants according to their animal hosts, this gene having a higher sequence variability compared to *groEL* and *msp4* [17, 22]. Both *msp2* and *msp4* belong to the OMP-1/MSP2/P44 superfamily [23]. The *msp4* sequence seems to be stable through the *A. phagocytophilum* life-cycle being a preferable genetic marker for phylogenetic analyses [22]. Sequences analysis showed a high degree of identity at the *msp4* locus, similar to the results using the *groESL* with the exception of roe deer strains, these being more diverse even than using *ankA* [22]. Different authors published studies of genetic variants using different terminology, such as ecotype (*groEL*), cluster (*ankA*) or genotype (*msp4*) [16, 20, 21]. “Ecotype” refers to hosts specificity of certain genotypes; “cluster” involves a deeper phylogenetic approach, while “genotype” is based on a purely genetic analysis. To refer to all mentioned terms, “genetic group” is used here. Different correlations of genetic variants have been found amongst vertebrate hosts, tick vectors and geographical locations. Infected humans, whether from Europe or America seem to share related strains belonging to the same genetic group [16, 20, 21]. Domestic animals like horses, dogs and cats, wild animals like red deer (*Cervus elaphus*), wild boars (*Sus scrofa*), red foxes (*Vulpes vulpes*) and hedgehogs (*Erinaceus* spp.) are harbouring strains with zoonotic potential related with human strains, while roe deer (*Capreolus capreolus*), rodents and birds seem to carry genetically distant strains [16, 19–21]. Regarding the strains infecting domestic ruminants, the studies present different results depending on the gene used for the analysis [16, 19–21] leading to some uncertainty about their possible involvement in the epidemiology of zoonotic infections. Further studies are necessary to establish which approach is discriminatory enough to discern between hosts with or without relevance to the epidemiology of HGA, especially since new highly discriminatory approaches such as multilocus sequence typing (MLST) and multiple-locus variable-number tandem repeat analysis (MLVA) are currently used [21, 24]. For instance, the MLST analysis on seven housekeeping genes (*pheS*, *glyA*, *fumC*, *mdh*, *sucA*, *dnaN* and *atpA*) revealed a similar pattern with *ankA* gene analysis, with strains from humans, dogs, horses, wild boar and hedgehogs belonging to the same clonal complex while other strains belonged to another seven clonal complexes [21]. The MLVA technique developed by Dugat et al. [24] showed the presence of slightly different profiles among the same host species (e.g. cattle) and different profiles between different hosts [24]. Based on this analysis, two epidemiological cycles were suggested for France, one involving red deer as reservoir hosts and domestic ruminants as either accidental or longer-term hosts, and another involving roe deer as reservoir hosts [24]. However, this study was based on a limited number of samples and a low variety of hosts, and further analysis could reveal the presence of multiple epidemiological cycles.

Despite the increasing number of studies on *A. phagocytophilum* genetic diversity, there are still insufficient data to understand the geographical distribution, host preferences and pathogenicity to humans of each described genetic variant. In this context, it is hard to analyse the relevance of these genetic groups for the public health. Moreover, despite several recent reviews, epidemiological data regarding human infections in Europe are poorly collated consisting of a collection of case reports and seroprevalence studies. The HGA epidemiology in Europe has not been critically reviewed. In this context, the aim of this review was to update epidemiological knowledge on European HGA, comparing this with what is known from the USA and to review diagnostic approaches.

## *Anaplasma phagocytophilum* (Foggie, 1949)

### The microorganism and its variability in Europe

*Anaplasma phagocytophilum* infection has been described under various acronyms according to the main species affected (TBF, EGA, CGA and HGA) [7, 11]. *Anaplasma phagocytophilum* infects mammalian neutrophils, where it replicates within cell membrane derived cytoplasmatic vacuoles named morulae [25, 26]. Morulae may contain one or more reticulate cells, dense cored cells, or both [26].

*Anaplasma phagocytophilum* has a single small circular chromosome (1.47 Mb) with abundant repeats (12.7%) that has been suggested to facilitate antigenic variation through recombination [15]. Of its 1369 open reading frames, 462 are unique encoding hypothetical, conserved hypothetical and conserved domain proteins, membrane proteins and lipoproteins [27–31]. Phylogenetic analyses of genes such as *groEL* [14–16], *ankA* [17–21] and *msp4* [22] of the European strains suggest the presence of different genetic variants and a correlation of these with the vertebrate hosts, tick vectors and also a possible correlation with geographical origin.

Regardless the gene used for analysis, infected humans, whether European or American, revealed the same genetic group [16, 20, 21]. Similarly, domestic animals like horses, dogs, and cats, share the same ecotype/cluster (I) with humans based upon phylogenetic analysis of *groEL* and *ankA* [16, 19–21]. Conversely, using a more discriminatory *ankA* gene analysis [32], revealed that dogs were infected with three different strains, one being the above-mentioned human variant and two different canine variants. Furthermore, *ankA* sequence analysis of infections of cattle, sheep and goats disclosed two strains belonging to clusters I and IV [19, 21]. Although *A. phagocytophilum* was also detected in others domestic animals, like donkeys [33], there are no data regarding the strain involved.

European wild ruminant infection displays yet further diversity of infecting ecotypes, clusters or genotypes including ecotype/cluster I [16, 19, 21]. *Anaplasma phagocytophilum* was detected in roe deer (*Ca. capreolus*) [19, 34], red deer (*Ce. elaphus*) [13, 35] and in Iberian red deer (*Ce. elaphus hispanicus*) [36]. It was also detected in fallow deer (*Dama dama*), sika deer (*Ce. nippon*) and Dybowskiʼs sika deer (*Ce. nippon hortulorum*), reindeer (*Rangifer tarandus*), elk (*Alces alces*), European bison (*Bison bonasus*), chamois (*Rupicapra rupicapra*), alpine ibex (*Capra ibex*), and mouflon (*Ovis musimon*) with variable prevalence [13, 24, 37, 38]. Among these, the red deer are considered one of the reservoir hosts for the human pathogenic strain, based on *groEL* sequence analysis belonging to ecotype I [16, 39]. Use of a more discriminatory *ankA* sequence revealed infection with strains belonging to cluster I and IV, and less in cluster III amongst red deer [19–21]. In contrast, roe deer seem to be infected mainly by strains belonging to *ankA* gene clusters II, III and less so by strains belonging to cluster IV [19–21] and by strains belonging to *groEL* ecotype II and a few strains belonging to ecotype I [16]. *AnkA* sequences from European bison and chamois mirror the strains found in red deer, belonging to clusters I and IV [19–21]. Whereas, mouflon share the same ecotype I with human strains based upon *groEL* analysis [16].

Limited reports have suggested wild boar (*S. scrofa*) as a potential reservoir hosts for *A. phagocytophilum* [40] with *ankA*, *groEL* and *msp4* gene analysis suggesting an overlap of clusters, ecotypes or genotypes with those of human significance [16, 21, 32].

Similarly to dogs, sequences obtained in wild carnivores, including red fox (*V. vulpes*), brown bear (*Ursus arctos*) and one timber wolf (*Canis lupus occidentalis*) cluster with human strains (cluster/ecotype/genotype I) using *ankA, groEL* and *msp4* sequences [16, 21, 32, 41].

Small mammals have been considered reservoir hosts for *A. phagocytophilum* [42] with infection reported in mice, voles and shrews [13, 16, 43–50], but their short life-span is likely to reduce their epidemiological importance as reservoir hosts, but this remains hotly debated [48]. Subsequent studies of *A. phagocytophilum* associated with *I. trianguliceps* and rodents, suggest distinct enzootic cycles [20, 44–47]. Infected voles and shrews revealed a distinct cluster V of *A. phagocytophilum* [20, 21]. There are also several reports regarding the occurrence of *A. phagocytophilum* infection in European hedgehogs (*Erinaceus europaeus*) [21, 51, 52], northern white-breasted hedgehog (*E. roumanicus*) [53] and black rat (*Rattus rattus*) [54]. Based on both *ankA* and *groEL* analysis, these hedgehog strains belonged to cluster I [16, 21]. In addition to these, *A. phagocytophilum* was also detected in European hares (*Lepus europaeus*) [55] and large rodents such as the crested porcupine (*Hystrix cristata*) [56] but phylogenetic data regarding these strains are limited.

A further lack of clarity surrounds infection of avian hosts with *A. phagocytophilum*. A distinct ecotype (IV) was reported in ticks collected from blackbirds (*Turdus merula*), suggesting the existence of a separate enzootic cycle for *A. phagocytophilum* in birds and probably utilizing *I. frontalis* ticks [16]. Nevertheless, birds are additionally important hosts for immature *I. ricinus*, which are also the main ticks biting humans [48], thus of public health relevance.

**Fig. 1:**
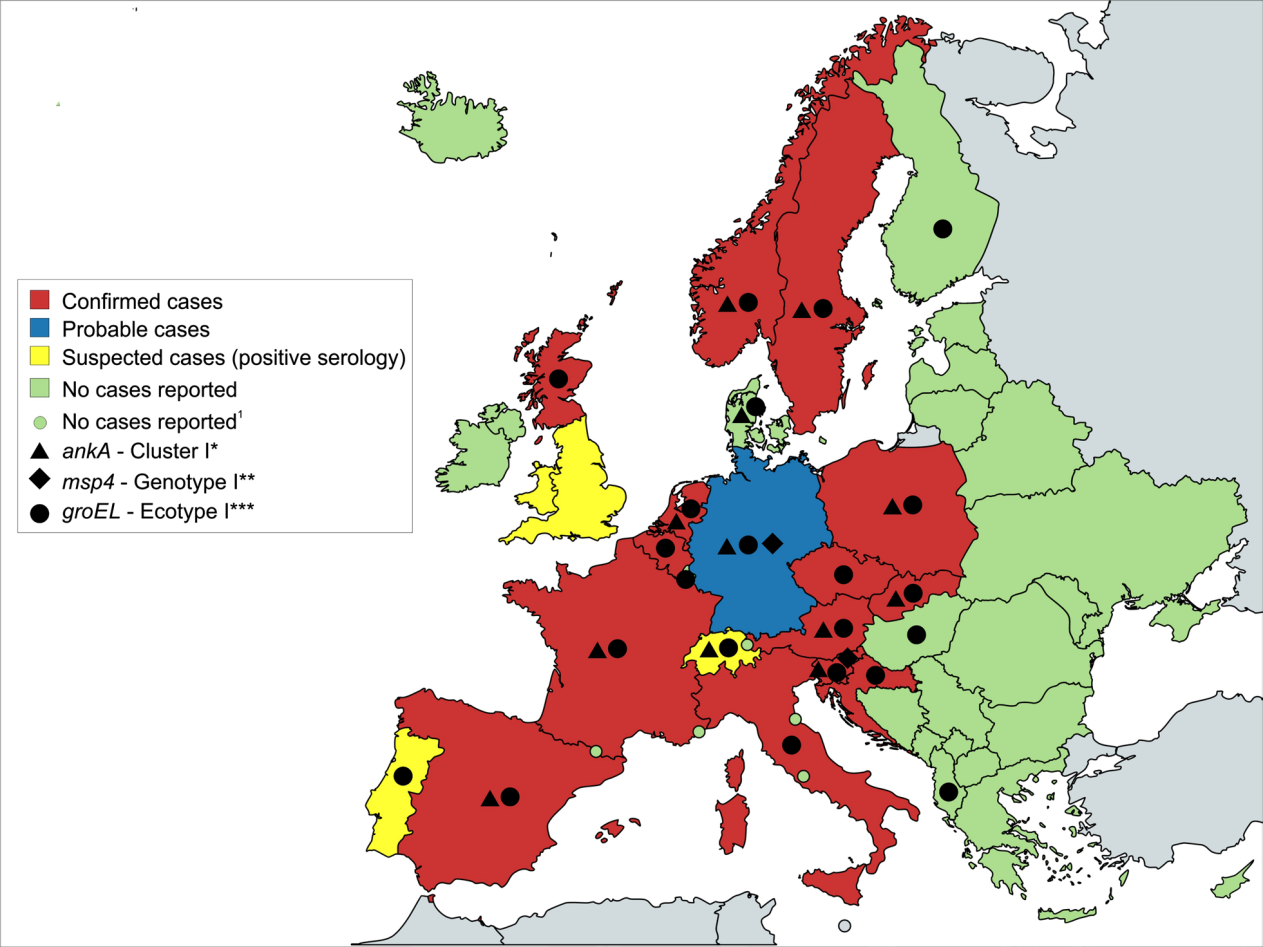
The geographical distribution of HGA cases and genetic groups including strains with zoonotic potential. *Notes*: ^1^Andora, Monaco, San Marino, Vatican; *Detected in various hosts (details presented in Table 1) [19, 21]; **Detected in various hosts (details presented in Table 1) [22, 32]. *** Detected in various hosts (details presented in Table 1) [16, 145]

The data on the geographical distribution of the summarized genetic variants are limited. The known geographical distribution of the genetic group containing strains with zoonotic potential is presented in Fig. 1. The hosts harbouring these strains and their geographical origin are presented in Table 1. Based on all these studies, the genetic group including human strains seems to be the most diverse and widespread [16, 17, 19–21]. In one of these studies the geographical distribution of each ecotype is presented [16]. In contrast with ecotype I, which seems to be spread in almost all Europe, the remaining ecotypes (II–IV) have a more limited distribution. Although their distribution overlaps with that of ecotype I [16], the limited distribution of the ecotypes II–IV may be influenced by the limited origin of the samples tested. In order to clarify the spread of each genetic group further studies should be performed.

**Table 1:** The hosts harbouring strains with zoonotic potential and their geographical origin

Common name	Scientific name	Origin	Genetic group (gene)	References
Bison	*Bison bonasus*	PO	Cluster I (*ankA*)	[19, 21]
Cow	*Bos taurus*	NO, EE		
Cat	*Felis catus*	AT, CH		
Chamois	*Rupicapra rupicapra*	SI		
Dog	*Canis familiaris*	CH, DE, DK, ES, FR, SE, SI, SK		
Hedgehogs	*Erinaceus europaeus*	DE		
Horse	*Equus caballus*	CH, DE, DK, NL		
Human	*Homo sapiens*	SI		
Red deer	*Cervus elaphus*	DE, PO, SI		
Red fox	*Vulpes vulpes*	DE		
Sheep	*Ovis aries*	DE, NO		
Wild boar	*Sus scrofa*	SI		
Brown bear	*Ursus arctos*	SI	Genotype I (*msp4*)	[22, 32]
Dog	*Canis familiaris*	SI		
Donkey	*Equus africanus asinus*	DE		
Horse	*Equus caballus*	DE		
Human	*Homo sapiens*	SI		
Wild boar	*Sus scrofa*	SI		
Alpine ibex	*Capra ibex*	AT	Ecotype I (*groEL*)	[16, 145]
Beech marten	*Martes foina*	BE		
Badger	*Meles meles*	BE		
Brown bear	*Ursus arctos*	HR, SI		
Caw	*Bos taurus*	FR, DE, NL, CH		
Cat	*Felis catus*	FI		
Chamois	*Rupicapra rupicapra*	AT, HR, SI		
Common blackbird	*Turdus merula*	CZ		
Dog	*Canis familiaris*	AL, HR, FI, DE, HU, IT, SI		
European hare	*Lepus europaeus*	HR		
European polecat	*Mustela putorius*	BE		
Fallow deer	*Dama dama*	DE, NL, SK		
Gray wolf	*Canis lupus*	HR		
Hedgehogs	*Erinaceus europaeus*	CZ, DE, HU		
Horse	*Equus caballus*	HR, CZ, FR, DE, IT, NL, SE		
Human	*Homo sapiens*	AT, BE, NL, PO, SI		
Mouflon	*Ovis musimon*	AT, HR, DE, NL, SK		
Mouse	*Alces alces*	NO, SE		
Red deer	*Cervus elaphus*	AT, HR, DE, NL, NO, PO, SI, ES		
Red fox	*Vulpes vulpes*	HR, DE, NL		
Red squirrel	*Sciurus vulgaris*	CZ		
Roe deer	*Capreolus capreolus*	HR, FR, DE, NL, PO, SI, CH		
Sheep	*Ovis aries*	HR, FR, NL, NO, GB		
Sika deer	*Cervus nippon*	DE		
Wild boar	*Sus scrofa*	HR, NL, SI, SK		
Wild goat	*Capra aegagrus*	DE, NL, GB, CH		
Only in vectors	*I. ricinus*	EE, LU, PT		

*Abbreviations*: AT, Austria; BE, Belgium; CZ, Czech Republic; DE, Germany; DK, Denmark; EE, Estonia; ES, Spain; FI, Finland; FR, France; GB, UK; HR, Croatia; HU, Hungary; IT, Italy; LU, Luxembourg; NL, Netherlands; PO, Poland; PT, Portugal; SE, Sweden; SI, Slovenia; SK, Slovakia; AL, Albania; CH, Switzerland; NO, Norway

This high diversity may be the result of an adaptation of *A. phagocytophilum* to different host species. Moreover, the co-infection of vectors with multiple genetic variants as it was suggested before in roe deer [57], may lead to the occurrence of new strains with different host preferences. The impact of strain heterogeneity on public health is not enough explored. However, the possible existence of independent enzootic cycles should decrease the pressure on human health.

In contrast with the heterogeneity of the European strains, American strains of *A. phagocytophilum* appear more restricted, primarily belonging to two variants (AP-ha and AP-V1), of which only AP-ha was detected in humans [13, 22]. However, a comparison between pathogenic and non-pathogenic strain diversity from the two continents is unsubstantiated since the hypothesis referring to non-pathogenic strains is not based on experimental data but on the lack of detection in humans.

In contrast with the European situation, in the USA human strains are maintained in nature through reservoir hosts such as white footed mice (*Peromyscus leucopus*), deer mice (*P. maniculatus*) and other rodents [13, 58, 59]. White-tailed deer (*Odocoileus virginianus*) are considered major reservoir hosts for variants which were never detected in humans, being suggested as non-pathogenic [13, 22]. Based on different markers (*ankA*, *groEL*, *gltA* and *msp4* genes), American and European human strains are grouped in different clades, being phylogenetically distinct [21, 22, 60]. However, based on *ankA* gene analysis, both European and American strains belong to the same genotype I, suggesting a degree of relatedness [19]. Whether differences in virulence and clinical manifestations observed between the American and European strains reside within their genetic composition or differences are driven by their eco-epidemiology remains to be resolved.

### Transmission and vectors

Transmission of *A. phagocytophilum* commonly occurs through the bite of an infected tick. *Ixodes ricinus* serves as the main vector in Europe [61, 62]. Transstadial transmission is important in maintaining *A. phagocytophilum* within its endemic cycles [6, 16, 49]. Although transovarial transmission has been suggested, its efficacy seems to be low [16, 49], necessitating further amplification by feeding upon reservoir species to maintain the bacteria in endemic cycles [49, 62]. *Ixodes ricinus* may become infected with *A. phagocytophilum* after feeding on an infected host, depending on various factors such as the percentage of infected neutrophils and the density of ticks feeding on the same host [63]. Co-feeding transmission from infected to uninfected ticks whilst feeding at common sites has not yet been reported for *Anaplasma* [64].

In addition to its main vectors, *A. phagocytophilum* has been detected in questing ticks belonging to other members of genus *Ixodes* including the European *I. persulcatus* [65], *I. trianguliceps* [66], *I. ventalloi* [67] and *I. hexagonus* [68]. Beyond *Ixodes*, *A. phagocytophilum* DNA has been detected in *Dermacentor reticulatus* [69], *Haemaphysalis punctata*, *H. concinna*, and *Rhipicephalus bursa* [70]. The vectorial capacity or these other European tick species has not been fully elucidated.

Despite regular detection of *A. phagocytophilum* DNA in *I. ricinus* in Europe, reports of infected ticks removed from humans are infrequent, being reported in Poland [71], Italy [49, 72], Romania [73] and the UK [74]. Among these, only in the UK study, the presence of *A. phagocytophilum* in two of the three ticks removed from a patient with non-specific clinical signs was demonstrated [74]. The patient developed clinical signs 3 days after the tick bite and was serologically diagnosed with HGA in accordance with CDC criteria by a 4-fold increase of *A. phagocytophilum*-specific IgG and IgM in paired serum samples collected at 8 and 28 days after tick removal [74]. The remaining studies only evaluate exposure risk [49, 71–73], rather than follow-up of those patients bitten by infected ticks. In the absence of patients’ follow-up, the results are difficult to interpret. However, the difference between high prevalence in ticks (e.g. 23.7% in Poland) and the patients not coming back for a medical consultation, together with the relative low number of reported cases, may be explained by a low transmission rate, asymptomatic cases or undiagnosed mild infection. Another suggested explanation for this discrepancy was the blood meal, which may trigger bacterial reactivation in infected ticks [71].

Beyond tick bite transmission, human infections have followed blood or red cell transfusions in both the USA and Europe [59, 75, 76]. Although only a single infection case of transfusion-acquired HGA has been described in Europe [75], several countries such as Poland and Belgium have reported blood donor seroprevalence to be high (5.4 and 14.5%, respectively), consequently the risk of infection *via* blood transfusion should be further investigated [77, 78].

Perinatal transmission from mother to child has only been described in the USA [79, 80]. The timing of neonatal infection was consistent with three potential transmission routes (intrauterine/transplacental, during the birth or through breast feeding); however, the transplacental route was suggested as being the most probable [81]. In Europe, transplacental transmission has been demonstrated in both cows and sheep [81, 82], and it was also suggested for dogs infected with a different *Anaplasma* species (i.e. *A. platys*) from Europe [83] and Africa [84].

Nosocomial exposure to HGA by direct contact with blood or respiratory secretion from a fatal HGA case was suggested only once in a Chinese hospital [85], but other authors contested the hypothesis, due to insufficient evidence [86]. Moreover, later it was confirmed that all patients had severe fever with thrombocytopenia syndrome virus (SFTSV) infection [87]. This agent, a newly discovered bunyavirus, causes a clinical picture which resembles previously described Chinese HGA cases [85, 87]. Subsequently, the possibilities of SFTSV and HGA co-infection or HGA misdiagnosis were debated in a series of comments and responses [88, 89]. In addition, by comparing the clinical picture of USA HGA cases with Chinese HGA cases and arguing the slight chances for simultaneous infection with both infectious agents, Wormser [90] impugned the accuracy of the diagnostics in reported Chinese cases.

## Human granulocytic anaplasmosis in Europe

### Geographical distribution and epidemiological indices

HGA was first diagnosed in 1990, in Wisconsin (USA) in a patient with tick bite history and severe febrile illness [11]. In Europe, the first human clinical case was described in Slovenia in 1997, but evidence of human infection pre-dated this back to 1995 in Switzerland and the UK [91–93]. Subsequently, HGA has been reported in several European countries (Fig. 1): Austria [94, 95]; Belgium [78, 96]; Croatia [97, 98]; Czech Republic [99, 100]; France [101, 102]; Germany [103]; Italy [22, 104]; Portugal [105]; the Netherlands [106]; Norway [107, 108]; Poland [109, 110]; Slovakia [111, 112]; Spain [113]; and Sweden [114]. The geographical distribution of *A. phagocytophilum* reported herein being based upon case reports, serological surveys or genetic studies.

The incidence of human HGA cases in Europe is lower (estimated under 300) than reported from the USA, where a steady increase has been reported since 2001, with more than 15,000 accumulated cases until 2015 [59]. This difference cannot be explained by pathogen prevalence in ticks as *A. phagocytophilum* is reported in some 3% of European *I. ricinus*, nearly as high as that among ticks in the USA [115]. On a cautionary note, the majority of studies do not provide sufficient data regarding the prevalence of each ecotype/genotype circulating in ticks and humans, potentially masking prevalence of potential zoonotic strains.

Human seroprevalence in Europe is on average ~ 8.3%, reaching up to 31% (Table 2). This incongruence between human seroprevalence and observed clinical cases might arise from incomplete diagnosis, or a high rate of asymptomatic infections [116], or serological cross-reactivity that might lead to an overestimation of seroprevalence rate [115]. This disparity is partially explained by Swedish studies in which more than half of the patients with an ongoing *A. phagocytophilum* infection (seroconversion or 4-fold increased antibody titre), failed to develop any other associated clinical symptoms upon follow-up interview, being defined as having subclinical infection [116].

**Table 2:** The seroprevalence of HGA in different tick exposure risk groups in Europe

Country	Location	Prevalence (%)	Total examined	Group	Method	References
Austria	Tyrol	2.62	191	TBD suspected	IFAT	[94]
Belgium	–	30.96	1350	TBD suspected	IFAT	[96]
Belgium	Walloon	14.20	148	Exposed workers	ELISA	[82]
	Namur	17.20	209	Blood donors		
	Brussels	14.50	193	Blood donors		
Czech Republic	Central Bohemia	15.15	66	EM patients	PCR; Seq	[100]
		18.18	66	EM patients	IFAT	
France	–	0.01	141,007		IFAT	[125]
	–	1.01	399		PCR	
France	Alsace	20.00	15		IFAT; PCR	[102]
France	Alsace	2.60	2908	Forest workers	ELISA	[123]
	Lorraine	1.30				
	Champagne-Ardenne	1.40				
	Bourgogne	1.00				
	Franche-Comté	2.30				
Germany	–	4.51	422	Ab to *B. burgdorferi*	IFAT	[104]
	–	1.20	249	Control group	IFAT	
Italy	–	6.33	79		IFAT	[105]
Norway	Telemark	10.34	58	LB patients	IFAT	[107]
		1.96	51	Control group		
Norway	Sogn og Fjordane	16.28	301	Blood donors	IFAT	[127]
Poland	Pulawy	26.10	46	Forest workers	IFAT	[122]
	Lubartów	35.90	39			
	Lublin	23.30	30			
	Sobibór	17.00	47			
	Zwierzyniec	23.60	55			
	Goscieradów	13.60	44			
	Lublin	5.4	56	Blood donors		
Poland	Lublin	20.63	63	Forest workers	IFAT	[119]
Poland	–	9.1	450	Endemic area	IFAT	[120]
		2	50	Blood donors		
Poland	Białystok	3.9	231	Forest workers	IFAT	[121]
Poland	Roztocze	17.7	113	Forest workers	IFAT	[76]
	Lublin	5.4	56	Blood donors		
Poland	–	10.91	110	TBE	PCR	[110]
Slovakia	–	25	76	TBD suspected	IFAT	[111]
Slovenia	–	60.87	46		PCR; Seq	[32]
Sweden	Koster Island	11.35	185		IFAT	[114]
Sweden	–	9.70	206	TBD suspected	IFAT	[116]
Switzerland	–	0.54	373	Newborns	IFAT	[81]
		1.13	530	Blood donors		
		8.91	258	Hunters		
		12.75	149	Ab to *B. burgdorferi*		
		19.51	205	Ab to TBE		

*Abbreviations*: Ab, antibody; TBD, tick borne disease; EM, erythema migrans; LB, Lyme borreliosis; Seq, sequencing

Reported seroprevalence appears highly variable, depending on the study, country, year and population included (Table 2). The majority of summarised studies refer to seropositive individuals in accordance with the probable case definition: serological evidence of elevated IgG antibody reactive with *A. phagocytophilum* antigen by IFA, with a cut-off of 1:64 (CDC case definitions of *Anaplasma phagocytophilum* infection). In the majority of these studies, serological testing was performed using commercial IFA kits utilising human isolates of *A. phagocytophilum* (different strains) cultivated in HL60 cells as antigen, with a cut-off value of 1:64. For the studies in which other serological assays or other criteria for interpretation were used, the details are provided.

Bakken et al. [107] compared HGA seroprevalence between Lyme borreliosis (LB) patients (study group) and healthy people (control group) in Norway. A total of 58 patients diagnosed with LB were tested for the presence of antibodies against *A. phagocytophilum* (at that time known as “*Ehrlichia equi*”) using *Ehrlichia equi* infected neutrophils as antigen. Values ≥ 1:80 were considered positive. The study group included patients with a presumed recent *I. ricinus* bite and serologically-confirmed active LB [107]. The results indicated that 10.34% of the patients were seropositive for both HGA and LB, showing that patients with LB were 5.28 times more likely to have had HGA than the control subjects [107]. Dumler et al. [114] published a similar survey on Koster Islands (Sweden). They tested randomly the population for the presence of HGA using the same protocol as described by Bakken et al. [107], and LB antibodies and found among the 21 HGA seropositive residents, six were seropositive also for LB [114]. Both these studies considered as seropositive patients with elevated antibody titer (≥ 1:80), having lower probability for non-specific reactivity compared to studies using lower titer (≥ 1:64). However, in both studies the results showed the presence of both HGA and LB antibody, without the confirmation of HGA, suggesting not necessarily a high probability of co-infection, but the increased contact risk with both pathogens. Since the vector is the same for both, the results indirectly showed an increase of seropositivity in individuals with high risk to tick exposure. This is also sustained by the results of an extensive study published by Pusterla et al. [117] involving 1515 individuals from Switzerland, stratified into groups according to their risk for tick exposure. Low risk groups included newborns, and randomly chosen blood donors with unknown tick exposure rate and a high-risk group comprised of hunters and those with other tick-borne infections. Serum samples were examined by IFA using a 1:80 cut-off value for antibodies against *A. phagocytophilum* (bovine leucocytes infected with “*Ehrlichia phagocytophila*” Swiss strain). Only 0.54% of the newborn samples had positive titres, potentially reflecting maternal antibodies, whereas 1.1% of blood donors were seropositive and for the high-risk group 9% seroprevalence in hunters; those with LB yielded 12.7%; whereas TBE cases revealed 19.5% seropositive for HE [117]. In addition to the studies in Norway and Sweden [107, 114], this study [117] showed high prevalence of HGA antibody in all tested high tick exposure risk groups, all suggesting the high exposure to ticks as a risk factor for HGA. The different seroprevalence between the tick exposed groups (hunters vs LB or TBE patients) may suggest also an increased risk for co-infection with other pathogens transmitted by *I. ricinus*. This is also sustained by other studies from other countries. In Slovakia, between 2002 and 2005, from 76 patients with a history of tick bite and symptoms resembling LB, 19 (25%) were seropositive, having ≥ 1:64 IgG antibody titer against *A. phagocytophilum*. Among these positives, 14 were additionally seropositive for LB [111]. In Germany, Kowalski et al. [104] conducted an 8-year (1994–2001) seroprevalence study in Berlin/Brandenburg, north-eastern Germany. They compared 422 sera from patients with a confirmed tick-bite (positive antibodies against *B. burgdorferi*) with 249 control sera positive for antibodies against a different spirochaete (*Treponema pallidum*) or against different obligate intracellular bacteria (*Chlamydia* spp.). As in other studies, among the LB antibody-positive specimens there were significantly more *A. phagocytophilum* antibody-positive samples (4.5%) than among controls (1.2%) [104]. However, without confirmations of HGA cases these results alone cannot confirm the hypothesis.

In addition to these serological data, three other studies described confirmed co-infections through seroconversion or DNA detection. An Italian study on 79 patients with tick bite history within 6 months and/or who were presented to hospital with a suspected tick-borne infection or aseptic meningitis yielded five cases (6%) with a positive HGA serology [118]. Among these, two were confirmed HGA cases (fever and seroconversion with a 4-fold change in serum antibody titer to *A. phagocytophilum*), one was a probable HGA case (fever and acute and convalescent serum samples with unchanging IFA titer), two patients had a possible HGA infection (serum samples with a titer of ≥ 1:128 at only the testing point), whilst three individuals had positive serology for LB. Moniuszko et al. [110] published a report on the presence of *A. phagocytophilum*, *Borrelia* and *Babesia* spp. DNA in the blood of 110 TBE (meningitis/encephalitis and positive serology) patients in Poland, comparing the results with a control group of 20 healthy blood donors. A prevalence of 10.9% *A. phagocytophilum*-TBEv co-infection was recorded and 2.7% for triple co-infection (TBEV–*Borrelia* sp.–*A. phagocytophilum*). Similarly, in Czech Republic, among 66 patients with erythema migrans (EM) twelve (all with positive PCR for *B. burgdorferi* (*s.l.*)) were seropositive by IFA IgG to HGA and ten (nine with positive PCR for *B. burgdorferi* (*s.l.*)) were PCR-positive from blood or skin samples [100]. Among 14 *A. phagocytophilum* and *B. burgdorferi* (*s.l.*) co-infected patients (confirmed by DNA detection), three were pregnant women; one subsequently aborted and the mother’s blood sample was positive for both *A. phagocytophilum* and *B. garinii* DNA. The two other women safely delivered, although one had *A. phagocytophilum-*positive blood and placenta, and the other *B. garinii-*positive skin, *A. phagocytophilum-*positive blood and *B. garinii-*positive placenta [100]. Despite the case confirmation through *A. phagocytophilum* DNA isolation, both these studies used *16S* rRNA gene fragment amplification and provided no data regarding the sequence analysis and the strain involved.

Similarly, high HGA seroprevalence was associated with occupational risks and/or populations living in endemic areas, with multiple cases reported. Tomasiewicz et al. [119] compared the HGA seroprevalence in 63 individuals with occupational exposure to tick bites (forest workers) and with tick bite history, with a blood donor control group (*n* = 30) from Poland. A seroprevalence of 20.6% was found for among the tick exposed group, with the vast majority (85%) also additionally having anti-*B. burgdorferi* antibodies. In contrast, none of the blood donors were seropositive. Grzeszczuk et al. [120] tested for HGA antibodies from 450 serum samples originating from north-eastern Poland (known to be endemic for LB and TBE) which were submitted for serological diagnosis of LB. The study included a control group comprised of 50 healthy blood donors. The HGA seroprevalence was 9.1% for people living in the endemic area, compared to 2% in healthy blood donors. A significant difference was found between forest workers (16.7%) and other occupational categories (4.6%). Similar serological findings were reported from other studies in Poland [77, 121, 122]. Cisak et al. [77] and Chmielewska-Badora et al. [122] reported a high seroprevalence (17.7% and 23%, respectively) in forestry workers compared with the control group consisting in healthy blood donors (5.4% in both studies) in Lublin region. Grzeszczuk et al. [121] reported a low seroprevalence (3.9%) in both forestry and office workers in Białystok vicinity [121].

In all Polish studies, a cut-off value of 1:64 was used, increasing the risk for non-specific reactivity, in this case the true seroprevalence being lower. Nevertheless, the differences between exposed and control populations may be still sustained by the obtained data. The low seroprevalence observed by Grzeszczuk et al. [121] compared with other studies [77, 122] may sustain the presence of endemic and non-endemic areas in Poland. However, despite these three studies using the same serology kit and cut-off value, a comparison between them is not possible since in the study by Grzeszczuk et al. [121] a control group was not tested. A low *A. phagocytophilum* seroprevalence was also reported in a survey on a high risk population (forestry workers, which are in general tick exposed) in north-eastern France, using an anti-*A. phagocytophilum* recombinant P44 antigen IgG ELISA, and IFA re-tested of doubtful or positive sera [123]. This ELISA technique was previously tested and showed a sensitivity of 87% at a 1:160 cut-off value and a specificity of 98%, being comparable to IFA procedures for the laboratory diagnosis of HGA [124]. From a total of 2908 forestry workers, only 1.7% were seropositive; however, regional variation with a higher seroprevalence (2.6%) was reported from Alsace [123]. This finding was consistent with previous findings according to which Alsace may be a focal endemic area [125]. During a ten-year study in France, involving 141,007 patients with a history of tick bite, sera were tested using a micro-immunofluorescence assay. Titres of ≥ 1:100 for IgG and ≥ 1:50 for IgM in acute phase serum and/or the presence of seroconversion were considered for the positive cases. Only one HGA case was diagnosed from 112,995 tested sera samples from 2000–2008, whereas five new confirmed cases of HGA among the 14,000 tested sera were identified in 2009 [125]. Similarly, from a total of 261 samples tested for *A. phagocytophilum* DNA using molecular diagnostic assays during 2000–2008, only one HGA case was diagnosed, whereas three new cases of HGA among the 81 samples were identified in 2009 [125]. All PCR confirmed HGA cases originated from Alsace, from where only nine samples were tested in total [125], highlighting the existence of focal endemic areas. Despite the amplification and sequencing of *16S* rRNA confirmed the infection, this conserved gene is not useful for genotyping, providing little information regarding the genetic variants involved in these cases.

In contrast with the suggested influence of diverse factors on the HGA seroprevalence, other studies seem to report no difference between the different risk categories. In Belgium, among 148 samples from workers who were professionally exposed to tick bites (veterinarians, farmers, hunters, and gamekeepers), 209 samples from rural blood donors and 193 samples from urban blood donors tested by IFA, a high *A. phagocytophilum* seroprevalence was observed, suggesting the presence of endemic areas in the country. Seroprevalence of *A. phagocytophilum* was estimated as 14.2% for the exposed workers, 17.2% for the rural blood donors, and 14.5% for the urban blood donors [78]. Even if a low cut-off value (1:64) was used, this high seroprevalence is sustained by another study from Belgium [96]. Among 1350 patients suspected of a tick-borne infection between 2000 and 2009, 418 (31%) of patients were found positive for either IgG or IgM antibodies, using IFA against *A. phagocytophilum*, for both IgG and IgM antibody (cut-off value 1:64 and 1:20, respectively) [96]. Among 322 serum samples available for confirmation, 111 fulfilled the case definition, namely history of tick bite, fever, and an at least a 4-fold increase in IgG titre [96, 126]. Similarly, in Norway, among 301 healthy blood donors, 49 (16.2%) were seropositive having an antibody titer higher than 80 [127]. The authors observed no significant difference according to gender, age, geography, self-reported number of tick bites or presence of antibodies to *B. burgdorferi* (*s.l.*) [127].

Based on these studies, countries with a greater risk highlighted by a high seroprevalence are Norway, Sweden, Germany, Belgium, Poland and Switzerland. The high HGA prevalence in co-infections with pathogens transmitted by the same tick vectors may be explained by simultaneous exposure. However, based on limited published data, a previous infection cannot be ruled out especially in non-confirmed cases through fever and seroconversion, or a 4-fold change in serum antibody titer to *A. phagocytophilum*, and/or a positive PCR.

### Clinical manifestation

Surprisingly few HGA cases have been reported from Europe, limiting reliable clinical description of these individuals. We reviewed the published data from the 76 patients in Europe for which clinical and laboratory data were available (Table 3) [111, 128, 129].

**Table 3:** Overview of clinical findings in HGA patients

Country	Age	Tb	I	Clinical signs	Laboratory findings	D	H	T	References
Austria	33	+	8	Fever, arthralgia, headache	Thrombocytopenia; increased levels of CRP, lactate dehydrogenase and bilirubin	S	+	+	[146]
	33	+	7	Fever, headache, sweats, splenomegaly	Thrombocytopenia; leucopenia; increased levels of CRP, AST/ALT, lactate dehydrogenase, neopterin; elevated erythrocyte sedimentation rate	S	+	+	[94]
	40	+	n	Fever, arthralgia, headache, sweats	Thrombocytopenia; leucopenia; increased levels of CRP, AST/ALT, lactate dehydrogenase, procalcitonin, neopterin; elevated erythrocyte sedimentation rate	S	+	+	
	63	+	8	Fever, arthralgia, headache, vertigo, sweats, splenomegaly	Thrombocytopenia; increased levels of CRP, lactate dehydrogenase, neopterin; elevated erythrocyte sedimentation rate	S	+	+	
	46	+	6	Fever, arthralgia, headache, sweats, splenomegaly	Thrombocytopenia; increased levels of CRP, AST/ALT, lactate dehydrogenase, neopterin; elevated erythrocyte sedimentation rate	S	−	−	
	32	−	n	Fever, arthralgia, headache, sweats	Thrombocytopenia; leucopenia; increased levels of CRP, AST/ALT, lactate dehydrogenase, procalcitonin, neopterin; elevated erythrocyte sedimentation rate	S	+	+	
	38	+	9	Fever, myalgia, arthralgia, headache	Increased levels of CRP and creatine kinase	P	+	+	[95]
France	47	−	n	Fever, myalgia, arthralgia, headache, sweats, cough (atypical pneumonitis)	Thrombocytopenia; leucopenia; increased levels of CRP, AST/ALT, lactate dehydrogenase and fibrinogen	P	+	+	[101]
Italy	33	+	n	–	nd	S	n	+	[118]
	69	+	n	–	nd	S	n	+	
	41	+	n	Fever, myalgia, arthralgia	nd	S	n	+	
	48	+	n	EM	nd	S	n	+	
	17	+	n	Fever, myalgia, arthralgia, headache, nucal rigidity, aseptic meningitis	nd	S	n	+	
Netherlands	58	−	n	Fever, chills, diarrhea	Thrombocytopenia; leucopenia; increased levels of AST/ALT	P	+	+	[106]
Poland	40	−	n	Fever, headache, hepatomegaly	Thrombocytopenia; increased levels of CRP, AST/ALT	P	+	+	[109]
	41	+	7	Fever, headache, meningeal signs, vertigo, weakness, hepatomegaly	Thrombocytopenia; increased levels of AST/ALT	S	+	+	
	22	+	7	Fever, headache, vomiting, abdominal pain, diarrhea, splenomegaly	Thrombocytopenia; leucopenia; increased levels of AST/ALT	P	+	+	
Scotland	40	+	3	−	Lactate dehydrogenase (248 U/L)	S	n	+	[74]
Slovenia	11	−	9	Fever, headache, conjunctivitis, erythematous throat, abdominal pain (right upper quadrant abdominal tenderness)	Thrombocytopenia; leucopenia; increased levels of CRP	P	+	+	[147]
	36	−	10	Tachypnea, tachycardia, hypotension, ARDS	Thrombocytopenia; increased levels of CRP, AST/ALT	P	+	+	[75]
	70	+	12	Fever, myalgia, arthralgia, headache, vomiting, conjunctivitis, lymphadenopathy	Thrombocytopenia; increased levels of CRP	P	–	–	[91]
	59	+	21	Fever, myalgia, arthralgia, headache, chills, vertigo	Thrombocytopenia; leucopenia; increased levels of CRP, AST/ALT	S	+	+	[148]
	43	+	7	Fever, myalgia, arthralgia, headache, chills, vertigo, dry cough (pneumonia)	Increased levels of CRP, AST/ALT	S	+	+	
	55	+	30	Fever, myalgia, arthralgia, headache, vertigo, vomiting	Thrombocytopenia; leucopenia; increased levels of CRP	P	−	−	
	36	+	15	Fever, arthralgia, headache, chills, vomiting, lymphadenopathy, hepatosplenomegaly	Thrombocytopenia; leucopenia; increased levels of CRP, AST/ALT	S	+	−	[149]
	22	+	n	Fever, myalgia, arthralgia, headache, conjunctivitis, EM	Thrombocytopenia; leucopenia; increased levels of CRP, AST/ALT	S	+	+	[150]
	63	+		Fever, myalgia, arthralgia, headache	Thrombocytopenia; leucopenia; increased levels of CRP, procalcitonin	P	+	+	[151]
	14	+	n	Fever, chills, neck and lumbar pain and passing of dark urine, jaundice and a discrete maculopapular rash on the trunk and neck, systolic murmur	Thrombocytopenia, leucopenia; increased levels of CRP, AST/ALT, lactate dehydrogenase, bilirubin	P	+	+	[152]
Spain	19	+	15	Fever, myalgia, headache, abdominal pain	Thrombocytopenia, leucopenia; increased levels of AST/ALT	P	+	+	[113]
Sweden	41	−	n	Fever, myalgia, headache, bilateral conjunctivitis, tachypnea, diffuse rash on the face and trunk, dry cough and dyspnea (bilateral interstitial infiltrates)	Increased levels of CRP, AST/ALT; intrahepatic cholestasis with increased serum bilirubin, serum alkaline phosphatase and serum glutamyl transpeptidase	P	+	+	[133]
	5	n	n	Fever, headache, facial palsy	nd	P	+	n	[132]
	41	n	n	Fever, headache, dyspnea, cough, conjunctivitis, rash	Increased levels of AST/ALT	P	+	+	
	32	+	30	Fever, headache, chills, stiff neck	Thrombocytopenia	P	+	+	

*Abbreviations*: Tb, history of tick bite; I, incubation (days); nd, not determined; D, diagnostic: S, serology; P, PCR; H, hospitalization; T, treatment received; n, not specified; −, no; +, yes

*Note*: the table is based on 33 cases

Age of patients varied between 5–70 years-old with a median of 53.5. Most of them (78.8%), recalled tick bite between 3–30 days (mean 12.7) before the onset of the disease, with most cases occurring between April and October. Determination of the duration and magnitude of bacteraemia in humans with HGA is challenging as laboratory examination is rarely undertaken during the early acute phase of infection. In a Slovenian study, the febrile period of the first five confirmed HGA cases had a mean of 7.5 days [130]. European HGA cases tend to present with mild or even asymptomatic infection, with complete recovery in two weeks, even in the absence of specific treatment [131]. Transient infection may occur in the absence of associated clinical signs; consequently, cases may not always be detected. However, among the patients included in this analysis, 62.8% were hospitalized, 73.1% received specific treatment and only in one report, two patients were asymptomatic. This discrepancy might relate to selective publishing bias with over-reporting of more severe clinical cases. Clinical presentation was usually as an acute non-specific febrile infection. Of those infected, 79.3% presented with pyrexia, 89% headache, 67.6% fatigue or malaise, 63.3% myalgia, 56.6% arthralgia and 39.2% with nausea. However, fever is more often reported. Considering the vast majority of the reports, the frequency of fever varies between 90–100%. One study [111], in which fever was reported in only 26.3% of the serologically confirmed cases can be considered doubtful since the authors refer to serologically confirmed cases but provide no data regarding the confirmation method. However, the authors report other clinical signs consistent with HGA infection and/or HGA and LB co-infections. Nevertheless, it is not clear if the reported cases are in acute or convalescent phase. Other clinical observation were: digestive signs (51.5%, including vomiting, diarrhea, abdominal pain, splenomegaly, hepatomegaly); exanthema/rash (23.8%); conjunctivitis (21.2%); lymphadenopathy (21.2%); cough (17.5%, including two cases of interstitial pneumonia, one atypical pneumonia, one case of ARDS-acute respiratory distress syndrome); neurological signs (15.5%, including vertigo in most of the cases, one case of facial palsy; one case of meningeal signs, one case of aseptic meningitis); and cardiac signs (tachycardia and hypotension in one case; one case of systolic murmur). The presence of erythema/rash was reported mainly in patients with LB or seropositive for *B. burgdorferi* (*s.l.*). However, there are reports in which co-infections are not specified [132] or are excluded [133]. In this last study, the patient developed a diffuse rash while acute and convalescent sera were negative for antibodies against *B. burgdorferi*, *Coxiella burnetii*, *Rickettsia conorii*, *R. typhi*, *Mycoplasma pneumoniae*, *Leptospira*, *Chlamydia pneumoniae* and *Ch. psittaci* [133]. Fatal infections are rare, but infection can cumulate in multi-system failure [59]. Co-infection with other tick-borne pathogens should be considered. Almost a third of HGA patients were additionally seropositive for *B. burgdorferi* (*s.l.*), less for *Ehrlichia chaffeensis* and two patients had concurrent TBE. The presumed co-infection with *E. chaffeensis* was not proven by DNA detection in these cases [129], suggesting a cross reaction. However, seroreactivity to *E. chaffeensis* in the absence of *A. phagocytophilum* antibody has been occasionally reported in the European human population [98], and one patient in Serbia was recognized to have clinical illness [134].

Cases acquired in Europe share the same clinical picture observed in USA, however, European cases are generally milder and thus far no fatalities have been reported. There is evidence of higher strain heterogeneity in Europe [21] that could correlate with host preference, pathogenesis and resulting virulence in humans [16]. This hypothesis is sustained by experimental infection in lambs with different *A. phagocytophilum* variants showing different pathogenic traits [135, 136]. In addition, factors related to different species of vectors may influence the virulence in an analogous situation as that between American and European *B. burgdorferi* strains [137].

### Diagnosis

According to the Centre for Disease Control and Prevention (CDC, Atlanta, Georgia, USA), confirmatory criteria for patients with consistent clinical presentations are either detection of *A. phagocytophilum* DNA in a clinical specimen *via* PCR amplification of a specific target, demonstration of *Anaplasma* antigen in a biopsy/autopsy sample by immunohistochemical methods, or isolation of *A. phagocytophilum* from a clinical specimen in a cell culture system. Serologically, a 4-fold change in antibody titre (IgG) against *A. phagocytophilum* antigen by IFA in paired (2–4 weeks) serum samples is confirmatory. Although for European HGA, there is no official case definition yet, both the European Centre for Disease Control and Prevention (ECDC) and the European Society of *Chlamydia*, *Coxiella*, *Anaplasma* and *Rickettsia*, formerly ESCAR: ESCMID Study Group on *Coxiella*, *Anaplasma*, *Rickettsia* and *Bartonella* (ESCCAR) guidelines are in concordance with the CDC guidelines.

Diagnosis of HGA should be based on clinical signs and patient’s history and can be supported by laboratory confirmatory tests. As described above, the symptoms of HGA may vary from patient to patient and can be difficult to distinguish from other conditions, especially other tick-borne diseases. Information such as recent tick bite, exposure to areas where ticks are likely to be found, or a history of recent travel to areas where HGA is endemic can be helpful in supporting the diagnosis. However, since *A. phagocytophilum* is endemic throughout Europe, the appropriateness of this latter criterion is limited. Routine blood tests, such as a complete blood cell count or a chemistry panel may be useful since thrombocytopenia, leukopenia or elevated liver enzyme levels are helpful predictors of anaplasmosis but may not be present in all patients. Common laboratory findings were: elevated CRP in 93.03%; elevated liver enzymes in 90% (alanine transaminase level; aspartate transaminase level); thrombocytopenia in 83.7% and leukopenia in 63%. Less commonly, in less than 50% of the cases the levels of lactate dehydrogenase and neopterin were increased, associated with elevated erythrocyte sedimentation rate and increased serum bilirubin [128].

Once clinically suspected, specialised laboratory testing should be undertaken for HGA confirmation. Indirect immunofluorescence using *A. phagocytophilum* whole antigen is often considered the gold standard serological test for diagnosis of HGA. Use of paired serum samples enables demonstration of a significant rise (4-fold) in antibody titres, using a cut-off value of at least 1:64 [126]. Ideally, the first sample should be collected in the first week of illness (during the acute phase) and the second and/or third between two to four weeks later [126]. IgM antibodies are less specific than IgG antibodies and are more likely to generate false positive results. Moreover, IgM results alone should not be used for laboratory diagnosis due to the low sensitivity [126, 138]. Serological tests based on enzyme immunoassay (EIA) technology are commercially available. IFA is generally used for screening and confirmation of HGA cases. The most commonly used commercial kit for IgG detection in the studies summarized in this review was from Focus Technologies, USA. According to the manufactures, the specificity of this test reaches 100%, and the sensitivity depends on the period between the moment of sampling and the beginning of the clinical signs, which ranges from 66.7% to 100% at a cut-off value of 1:64. Similarly, other IFA IgG kits have an 80–86.6% sensitivity and 92.7% specificity [126]. The ELISA technique was used only in few reports. The performance characteristics were evaluated by Ijdo et al. [124], showing an 87% sensitivity at a 1:160 cut-off value and a specificity of 98%, being comparable to IFA procedures [124]. However, this technique has been used in a limited number of studies providing insufficient support for routine use in diagnostic laboratories.

Acute phase whole blood samples can be tested by PCR targeting various genes such as *16S* rRNA or *msp2* [139]. This method is most sensitive in the first week of illness, but rapidly decreases in sensitivity following the administration of appropriate antibiotics. The analysis of published HGA cases in Europe (see above) have shown a relative low percent 68.2% of positive PCR results. Similarly, among 46 Slovenian confirmed cases of human anaplasmosis compatible with ESCCAR guidelines, only 28 (60.9%) of them were positive for the presence of *A. phagocytophilum* DNA [139]. Thus, a positive PCR result may be helpful, but a negative result does not exclude the diagnosis, and treatment should not be withheld due to a negative PCR result. In addition to acute phase sample collection time, the sensitivity of molecular detection also depends on: (i) sample type and quality, full blood or buffy coat being considered the more suitable compared with plasma [139] because of the tropism of *A. phagocytophilum* for white cells; and (ii) the number of genomic target gene copies and the amplicon length (short sequences being generally preferred to long ones for screening; longer ones being more used for sequencing and phylogenetic analysis) [139]. Most frequently used target genes for *Anaplasma* spp., include *16S* rRNA (*rrs*), heat-shock protein (*groEL*), citrate synthase (*gltA*), and major surface proteins (*msp1, msp2, msp4, msp5*). For molecular screening, the sensitive multicopy *msp2* is particularly useful, whereas for sequence comparison and database crossmatch, conservative or moderately conservative *rrs* and *groEL* strategies are regarded as a better choice [139].

During the first week of illness, a microscopic examination of blood smears may reveal morulae of *A. phagocytophilum* in the cytoplasm of the neutrophils. However, the percent of patients presenting intracytoplasmic morulae in the acute phase may vary from low values in Europe [129] to high values of 25–60% or even more in the USA [140, 141]. Although sensitivity is limited, this can be improved if the smear is performed from the buffy coat [139]. Gram staining is not suitable to visualize intracellular bacteria because of a lack of contrast against the host cytoplasm. Romanowsky staining is generally used, usually with a quick method such as Diff-Quik. This approach stains the bacteria purple, which allows the visualization of characteristic morulae. Morulae are usually 1.5–2.5 µm in diameter but can be as large as 6 µm [140].

Similar with the DNA detection, *in vitro* cultivation may be used also in the acute phase of illness. Cultivation of *A. phagocytophilum* from human blood has been used since 1996, when Goodman et al. [142] successfully isolated the bacterium on HL-60 cells. More recently, cultivation from blood was also successfully achieved from two patients from Czech Republic [100].

### Treatment

Chemical prophylaxis is not recommended after a tick bite, even in endemic regions [131]. Doxycycline is considered the drug of choice with good results for HGA in adults as well as in children older than eight years. Treatment should be instituted on clinical suspicion alone to avoid the potential for serious complication, [59]. Doxycycline (100 mg twice daily by IV or PO between 10–14 days) is highly effective and post-therapeutic relapses have not been reported [59, 143]. There is generally a rapid response to treatment with a marked clinical improvement within 24–48 h [59]. A possible alternative for children and patients with a doxycycline allergy or pregnant women is rifampicin with the following dose: for children 20 mg/kg/day, maximum 600 mg in two doses PO and for adults 300 mg, twice 2 times daily PO for 5–7 days in both cases [59, 144]. Other antibiotics, such as quinolones, cephalosporin’s, penicillin’s, and macrolides are ineffective [143]. To prevent infection, precautions should be taken to avoid exposure to ticks.

## Gaps remaining

Despite the great efforts of researchers for a better characterisation of HGA and *A. phagocytophilum* in Europe, there are several gaps remaining. The majority of them are related with the ecology and genetic diversity and their correlation with the pathogenicity.

First of all, it is important to be established how much the terms of different genetic variants (e.g. clusters, ecotype or genotype) are overlapping. The authors used different terms for these variants according to the gene analysed or maybe to their own preferences. However, for a better understanding of the pathogen genetic variability it is necessary to reach a consensus.

Regarding the pathogenicity to humans, it is not clear if strains less related with the human isolates, belonging to different genetic groups (e.g. rodent, bird, or roe deer strains in Europe or AP-V1 in USA) were not detected in humans because they are non-pathogenic, or because they cause asymptomatic infections. One important question is if they cause or not a serological response.

Similarly, the strains belonging to the same genetic group as human strains have zoonotic potential; however, it is not clear whether they have a different pathogenic potential for humans. Regardless of the study, the genetic group including human strains is the most diverse, clustering together strains from a large variety of hosts (Table 1). However, depending on the gene used, some strains (detected in sheep, goats, cows, hedgehogs, wild carnivores etc.) may belong to different groups. Because of this it is important to be established which approach is discriminatory enough to evaluate the risk for human health. Additionally, in order to evaluate the public health risks, the prevalence and geographical distribution of each genetic group should be further evaluated.

Another important but insufficiently clear aspect is the understanding of differences between HGA in USA and Europe. Although, there are clear differences between the ecology of American and European strains (e.g. different vectors, different hosts, apparently different genetic variability), it is not clear if the ecology or the genetic differences alone influence the pathogenicity to humans, or whether this may influence the prevalence of infections and increase the risk for developing more severe forms of diseases. Even more unclear, and therefore an important topic for future research, is tackling the differences in both pathogenicity and ecology between European and Asian strains.

Other gaps are related to the diagnosis and the seroprevalence or prevalence of HGA. Since HGA in Europe is not a disease with a mandatory surveillance and reporting, some cases may be not published. Moreover, clinical suspicion or even serological detection may be not be followed by confirmatory tests but may be treated. In addition, the unspecific clinical picture may lead to underdiagnosing. In this case, the prevalence is estimated based on published data and thus influenced by researchersʼ interest and by the approach they used. In addition, in the absence of mandatory surveillance and an official case definition in Europe, the diagnostic approaches may differ between the laboratories. In this case, interpretation of results interpretation and classification of suspected, probable and confirmed cases should be made with caution. Even following supportive and/or confirmed laboratory criteria published by CDC, (e.g. IFA IgG with a cut-off of ≥ 1:64 as supportive criteria, or detection of *A. phagocytophilum* DNA in a clinical specimen *via* amplification of a specific target in a PCR assay as confirmatory criteria), the published cases can be questioned if a single approach is used, especially if low titer of < 640 is obtained or if a single target gene is amplified but sequenced.

## Conclusions

Despite the apparently ubiquitous presence of *A. phagocytophilum* in ticks and various wild and domestic animals from Europe, published clinical cases of HGA remain rare, currently only a few hundred. It is unclear if this reflects the incidence of human infection in Europe or if the disease is underdiagnosed or underreported. Epidemiologic studies in Europe have suggested an increased occupational risk of infection for forestry workers, hunters, veterinarians, and farmers with a tick-bite history and also those living in endemic areas. Another risk factor for HGA seems to be infection with other pathogens transmitted by *I. ricinus*, mainly *B. burgdorferi* (*s.l.*). Although the overall genetic diversity of *A. phagocytophilum* in Europe seems to be higher than in the USA, the strains responsible for human infections are related on both continents, hence a difference in pathogenicity seems unlikely. However, to date, the study of the genetic variability and assessment of the difference in pathogenicity and infectivity between strains to various hosts has been insufficiently explored.

## Data Availability

Not applicable.

## Abbreviations

*ankA*: gene encoding a cytoplasmic protein antigen with ankyrin repeats
ARDS: acute respiratory distress syndrome
CDC: Centers for Disease Control and Prevention
CGA: canine granulocytic anaplasmosis
CRP: C-reactive protein
DNA: deoxyribonucleic acid
EGA: equine granulocytic anaplasmosis
EIA: enzyme immunoassay
ELISA: enzyme-linked immunosorbent assay
EM: erythema migrans
ESCAR: ESCMID Study Group on *Coxiella*, *Anaplasma*, *Rickettsia* and *Bartonella*
ESCCAR: European Society of *Chlamydia*, *Coxiella*, *Anaplasma* and *Rickettsia*
*groEL*: chaperone protein encoding gene
HGA: human granulocytic anaplasmosis
IFA: immunofluorescence assay
IgG: immunoglobulin G
IgM: immunoglobulin M
IV: intravenous
LB: Lyme borreliosis
*msp1, 2, 3, 4*: genes encoding major surface protein 1, 2, 3, and 4
MLST: multilocus sequence typing
MLVA: multiple-locus variable-number tandem repeat analysis
P44: protein p44
PCR: polymerase chain reaction
PO: *per os* (oral therapy)
RNA: ribonucleic acid
*rrs*: *16S* rRNA (*16S* ribosomal RNA gene)
*s.l.*: *sensu lato*
TBE: tick borne encephalitis
TBEv: tick borne encephalitis virus
TBF: tick-borne fever
TBP: tick-borne pathogen
